# Food patch use of Japanese quail (*Coturnix japonica*) varies with personality traits

**DOI:** 10.1186/s12983-023-00510-2

**Published:** 2023-08-31

**Authors:** Chunlin Li, Xinyu Zhang, Lin Cheng, Baowei Zhang, Feng Zhang

**Affiliations:** 1 School of Resources and Environmental Engineering, Anhui University, No.111, Jiulong Road, Hefei, 230601 China; 2 Anhui Province Key Laboratory of Wetland Ecosystem Protection and Restoration, Anhui University, No.111, Jiulong Road, Hefei, 230601 China; 3 Anhui Shengjin Lake Wetland Ecology National Long-Term Scientific Research Base, Dongzhi, 247230 China; 4 Anhui Vocational and Technical College of Forestry, No. 99, Yulan Road, Hefei, 230031 China; 5 School of Life Sciences, Anhui University, No.111, Jiulong Road, Hefei, 230601 China; 6 School of Statistics and Mathematics, Yunnan University of Finance and Economics, Kunming, 650221 China

**Keywords:** Animal personality, Food patch use, Foraging decisions, Japanese quail

## Abstract

**Background:**

The classic optimal foraging theory (OFT) predicts animals’ food patch use assuming that individuals in a population use the same strategy while foraging. However, due to the existence of animal personality, i.e. repeatable inter-individual differences and intra-individual consistency in behaviours over time and/or across contexts, individuals often exhibit different behavioural strategies, challenging the basic assumptions of the OFT. Here, we tested whether personality traits (boldness and exploration in open arena) of Japanese quail (*Coturnix japonica*, 38 females and 34 males) influenced their patch use in two foraging experiments with different inter-patch distances (i.e. 2 m in Experiment 1 and 3 m in Experiment 2).

**Results:**

The total feeding time and food intake of individuals did not differ between Experiment 1 and 2, but in both experiments, proactive (i.e. bolder and more explorative) individuals had longer feeding time and higher food intake than reactive individuals. In Experiment 1, proactive quails changed patches more frequently and had shorter mean patch residence time than reactive individuals, while the effects were not significant in Experiment 2. The quails reduced patch residence time along with feeding, and this trend was weakened in Experiment 2 which had longer inter-patch distance.

**Conclusions:**

The above results suggest that personality traits affect animals’ patch use, while the effects might be weakened with longer inter-patch distance. Our study highlights that animal personality should be considered when investigating animals’ foraging behaviours because individuals may not adopt the same strategy as previously assumed. Furthermore, the interaction between personality traits and inter-patch distances, which is related to movement cost and capacity of information gathering, should also be considered.

## Background

Foraging is directly linked to the survival of animals and has always been a hot topic in behavioural ecology [1, 2]. Exploring the foraging pattern and the influencing factors can help understand how animals increase their fitness in various environments [3]. Due to its critical significance, foraging behaviour has a long history of theoretical and empirical studies, the most well-known of which is the classic optimal foraging theory (OFT) [4]. The OFT suggests that animals should adopt optimal foraging strategies to increase food consumption while reducing foraging costs, which is favoured by natural selection [5].

For food patch use, the OFT predicts that an animal would leave a patch when its rate of food intake in that patch drops to the average rate of the habitat [5]. The patch use behaviour is a part of foraging decisions which are largely correlated with food gains and social interactions [6]. Food is usually patchily distributed, and animals exploring new patches are expected to have higher cost when travelling between patches. However, an individual’s food intake rate in a patch would decrease along with its foraging. To maximize their food intake, animals should make decisions on food types, patch types, time spent in patches (residence time), and between-patch movement [5, 7]. The predictions of OFT are based on the assumption that individuals within a population use the same strategy [8]. Specifically, individuals are assumed to allocate the same time foraging within a certain food patch and have the same optimal departure time [9, 10]. In these studies, individual differences in patch use may exist but are considered to be random variations around an optimal behaviour exhibited by each individual within a population [11]. However, consistent inter-individual behavioural differences have been found in a wide range of animal species and may challenge the basic assumptions of the OFT.

The consistence of behavioural differences among individuals within a population across time and/or contexts is defined as animal personality which has been found in a wide range of vertebrates and invertebrates during the last few decades [12]. As proposed by Réale et al. [13], animal personality is normally determined by quantifying repeatability of behavioural traits, such as boldness, exploration, activity, aggressiveness and sociability, which are commonly measured by testing single or multiple behaviours. Along with the increasing evidence of animal personality, its ecological and evolutionary significance has rapidly become a research focus [14]. The existence of personality may limit flexibility of animals’ behavioural responses and thus should be seriously considered in behavioural studies.

The five commonly measured personality traits might be linked to animals’ foraging behaviour. Among these traits, boldness measures animal’s willingness to take risks in novel environments; exploration measures its exploration of a novel object or a novel environment [15]. These two behavioural traits are often positively correlated and thus we can characterize individuals on the proactive–reactive axis [16]. Previous studies have suggested that boldness and exploration might influence the time to process new information, the ability to locate new resources and the response to uncertainty [17, 18]. Therefore, the proactive–reactive level of animals may be linked to many aspects of their foraging behaviour [19, 20]. For example, bolder chacma baboons (*Papio ursinus*) can locate food resources faster in new environments [20]. More exploratory great tits (*Parus major*) may perform better in finding food because they cover more places and accumulate more information [21]. The effects of personality traits in finding food may also covary with distances between possible food patches. As the between-patch distances increase, the movement cost and the risk of starvation and being preyed upon increase if an animal leaves the current patch to find food in a next one [22–24]. Also, the animal’s perception of whether the next patch has food of higher quality decreases with distances because of the difficulty of requiring reliable information [25, 26]. These costs may create dilemma for animals, that is, whether to find new patches with potentially abundant food or to continue feeding in the current patch with decreasing food intake rate (the exploration–exploitation trade-off) [26]. Individuals at different positions of the proactive–reactive axis may vary in their decisions on the exploration–exploitation trade-off. Proactive ones may try to find new patches with abundant food, while reactive ones might stay in the current patch, especially when inter-patches distances are long [26]. Although there are some studies reporting that boldness and/or exploration might be related to animals’ foraging behaviour, such as foraging tactics, diet, foraging locations and food consumptions [27, 28], no studies have investigated their joint effects on pattern of food patch use under different between-patch distances.

In this study, we tested whether boldness and exploration of domestic Japanese quail (*Coturnix japonica*) influenced their patch use and the resultant food intake under different between-patch distances. We first measured quails’ boldness and exploration using open arena assays (the same data reported in Zhang et al. [29]) and then quantified their food patch use in two foraging experiments with different distances between food patches (i.e. 2 m in Experiment 1 and 3 m in Experiment 2). Since proactive (i.e. bolder and more explorative) individuals have a faster pace-of-life requiring more food to maintain their higher metabolic rates [30], we expected that proactive quails would have longer feeding time and higher food intake. Because proactive individuals are more likely to take risks and explore more in novel environments [26, 31], we hypothesized that proactive quails would have more frequent shifts between food patches, and shorter residence time in each patch. Because distance between food patches is positively related to movement cost and affects information gathering [25], we predicted that the effects of personality traits would be weakened in Experiment 2 which had a longer inter-patch distance. In addition, because animal behaviour might be correlated with sex and body weight, we also tested the effects of these two factors on patch use of Japanese quail.

## Results

**Fig. 1:**
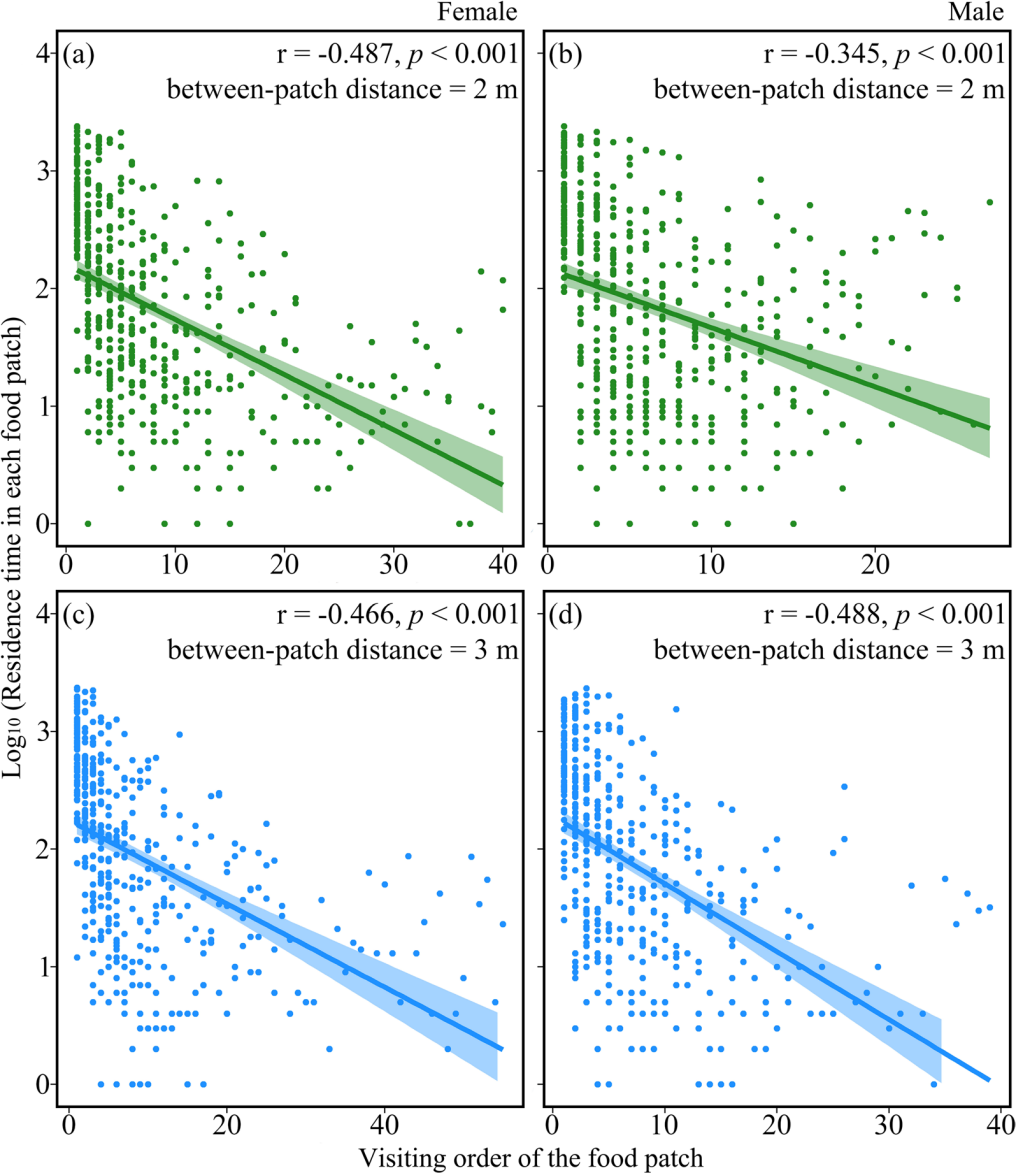
Correlations between residence time in each food patch and its order of being visited in the two patch use experiments with different between-patch distances (i.e. 2 m in Experiment 1 and 3 m in Experiment 2): **a** female quails in Experiment 1, **b** male quails in Experiment 1, **c** female quails in Experiment 2, and **d** male quails in Experiment 2

In total, we tested 72 individuals (38 females and 34 males) in this study and the average weight was 100.1 ± 6.8 g and 113.3 ± 10.0 g for females and males, respectively. The residence time in each food patch was negatively correlated with its order of being visited in the two patch use trials (Fig. 1).

**Fig. 2:**
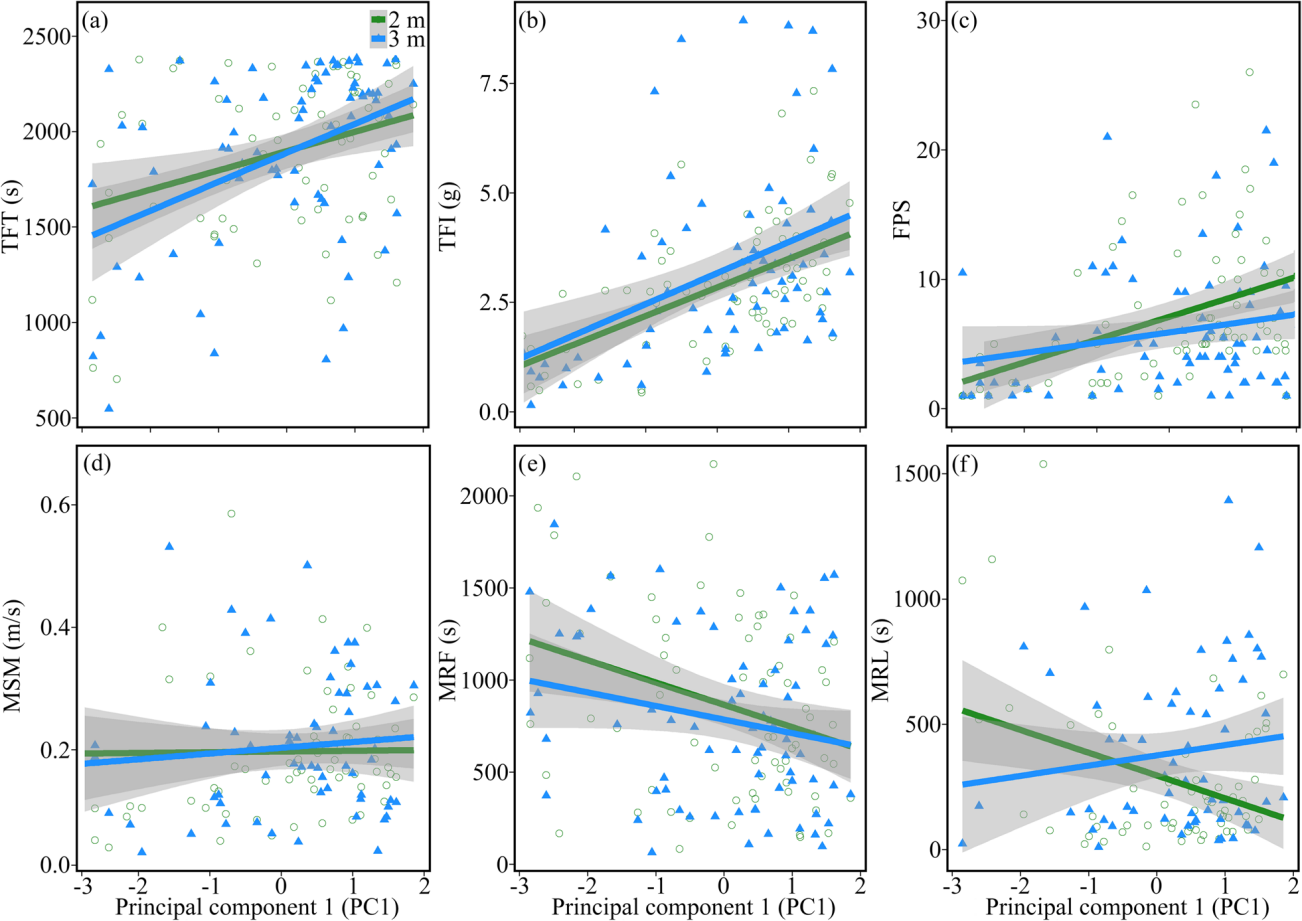
The relationship between the first principal component (PC1) of personality traits (proactivity) and the six foraging variables in the two patch use experiments with different between-patch distances (i.e. 2 m in Experiment 1 and 3 m in Experiment 2): total feeding time (TFT, **a**), total food intake (TFI, **b**), frequency of patch shifts (FPS, **c**), mean speed of inter-patch movement (MSM, **d**), mean residence time during the first (MRF, **e**) and last half of the feeding trial (MRL, **f**)

The average of the total feeding time was 1898.8 ± 411.5 s in Experiment 1 and 1889.7 ± 465.6 s in Experiment 2 (Table 1). It was positively correlated with proactivity in both Experiment 1 and 2 (Tables 2, 3 and Fig. 2a). The average of the total food intake was 2.8 ± 1.5 g in Experiment 1 and 3.2 ± 2.1 g in Experiment 2 (Table 1). It was positively correlated with proactivity in both experiments (Tables 2, 3 and Fig. 2b). The average of the frequency of patch shifts was 7.1 ± 5.5 in Experiment 1 and 6.4 ± 6.2 in Experiment 2 (Table 1). It was positively correlated with proactivity in Experiment 1 (Tables 2, 3 and Fig. 2c). The mean speed of inter-patch movement (MSM) was 0.18 ± 0.11 m/s in Experiment 1 and 0.19 ± 0.11 m/s in Experiment 2 (Table 1). There were no significant effects on MSM in either experiment (Tables 2, 3 and Fig. 2d). The mean residence time during the first half of the feeding trial was 866.2 ± 503.9 s in Experiment 1 and 786.4 ± 457.8 s in Experiment 2 (Table 1). It was negatively correlated with proactivity in Experiment 1 (Tables 2, 3 and Fig. 2e). The mean residence time during the last half of the feeding trial was 279.0 ± 296.4 s in Experiment 1 and 388.5 ± 330.5 s in Experiment 2 (Table 1). It was negatively correlated with proactivity in Experiment 1 (Tables 2, 3 and Fig. 2f). Sex and body weight had no significant effects on any of the above six foraging variables (Tables 2 and 3).

**Table 1:** The average scores (± SD) of the six foraging variables for Japanese quails in the two patch use experiments: total feeding time (TFT), total food intake (TFI), frequency of patch shifts (FPS), mean speed of inter-patch movement (MSM), mean residence time during the first (MRF) and last half of the feeding trial (MRL)

Foraging variables	Experiment 1		Experiment 2	
	Female	Male	Female	Male
TFT (s)	1895.3 ± 432.2	1902.7 ± 393.6	1841.0 ± 476.4	1944.1 ± 454.0
TFI (g)	2.6 ± 1.5	3.1 ± 1.4	3.1 ± 2.5	3.2 ± 1.7
FPS	6.8 ± 5.8	7.5 ± 5.1	6.0 ± 7.1	6.7 ± 5.2
MSM (m/s)	0.17 ± 0.10	0.18 ± 0.13	0.20 ± 0.12	0.18 ± 0.10
MRF (s)	868.2 ± 524.1	864.0 ± 488.1	792.5 ± 464.1	779.5 ± 457.6
MRL (s)	301.8 ± 339.2	253.9 ± 244.1	420.9 ± 315.0	356.1 ± 347.6

**Table 2:** The effects of proactivity, body weight and sex on the six foraging variables in Experiment 1 (between-patch distance = 2 m): total feeding time (TFT), total food intake (TFI), frequency of patch shifts (FPS), mean speed of inter-patch movement (MSM), mean residence time during the first (MRF) and last half of the feeding trial (MRL)

	Factors	Coefficient	S.E.	t value	*p* value
TFT (s)	Proactivity	121.026	38.283	3.161	**0.002**
	Body weight	8.006	5.698	1.405	0.165
	Sex	62.910	117.148	0.537	0.593
TFI (g)	Proactivity	0.676	0.118	5.708	** < 0.001**
	Body weight	0.016	0.018	0.928	0.357
	Sex	0.394	0.363	1.086	0.281
FPS	Proactivity	1.730	0.494	3.505	**0.001**
	Body weight	− 0.013	0.073	− 0.180	0.857
	Sex	− 0.161	1.511	− 0.107	0.915
MSM (m/s)	Proactivity	− 0.007	0.012	− 0.541	0.591
	Body weight	− 0.003	0.002	− 1.456	0.151
	Sex	− 0.026	0.036	− 0.730	0.468
MRF (s)	Proactivity	− 117.400	47.585	− 2.467	**0.016**
	Body weight	3.035	7.082	0.429	0.670
	Sex	85.055	145.613	0.584	0.561
MRL (s)	Proactivity	− 87.707	33.400	− 2.626	**0.011**
	Body weight	0.884	4.535	0.195	0.846
	Sex	− 22.469	90.958	− 0.247	0.806

Significant effects (*p* < 0.05) are displayed in bold

**Table 3:** The effects of effects of proactivity, body weight and sex on the six foraging variables in Experiment 2 (between-patch distance = 3 m): total feeding time (TFT), total food intake (TFI), frequency of patch shifts (FPS), mean speed of inter-patch movement (MSM), mean residence time during the first (MRF) and last half of the feeding trial (MRL)

	Factors	Coefficient	S.E.	t value	*p* value
TFT (s)	Proactivity	134.161	41.605	3.225	**0.002**
	Body weight	− 6.755	6.192	− 1.091	0.279
	Sex	− 42.338	127.314	− 0.333	0.740
TFI (g)	Proactivity	0.723	0.189	3.832	** < 0.001**
	Body weight	0.001	0.028	0.042	0.967
	Sex	− 0.196	0.578	− 0.339	0.736
FPS	Proactivity	0.763	0.608	1.254	0.214
	Body weight	− 0.049	0.091	− 0.539	0.592
	Sex	− 0.255	1.861	− 0.137	0.891
MSM (m/s)	Proactivity	0.000	0.013	0.000	1.000
	Body weight	− 0.002	0.002	− 1.363	0.178
	Sex	− 0.056	0.036	− 1.560	0.124
MRF (s)	Proactivity	− 59.397	44.252	− 1.342	0.184
	Body weight	7.035	6.586	1.068	0.289
	Sex	104.930	135.416	0.775	0.441
MRL (s)	Proactivity	34.562	42.939	0.805	0.424
	Body weight	− 2.396	5.309	− 0.451	0.654
	Sex	− 90.490	108.383	− 0.835	0.407

Significant effects (*p* < 0.05) are displayed in bold

**Fig. 3:**
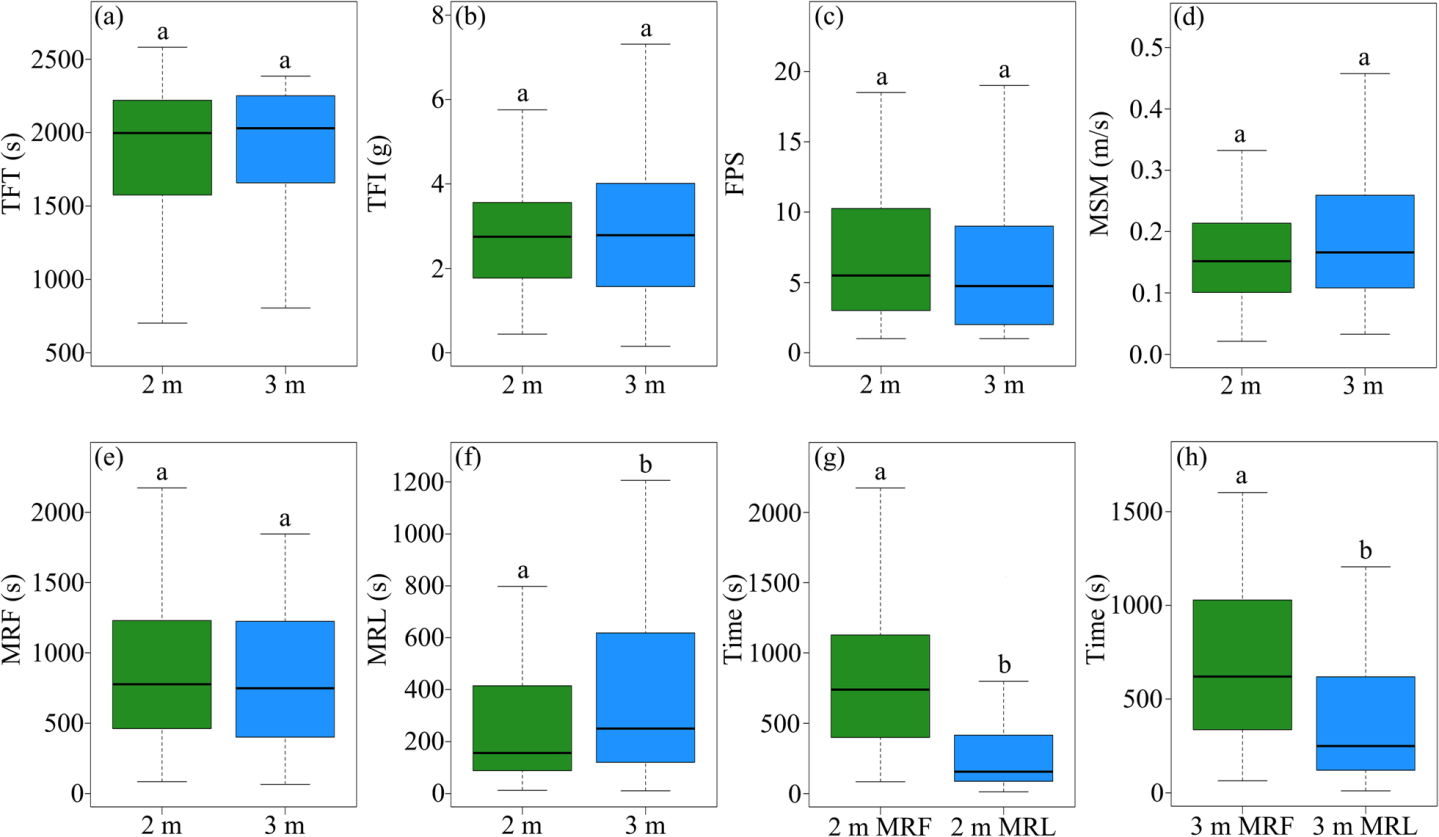
The differences in the six foraging variables between the two patch use experiments with different between-patch distances (i.e. 2 m in Experiment 1 and 3 m in Experiment 2): total feeding time (TFT, **a**), total food intake (TFI, **b**), the frequency of patch shifts (FPS, **c**), mean speed of inter-patch movement (MSM, **d**), mean residence time during the first (MRF, **e**) and last half of the feeding trial (MRL, **f**). The differences in mean residence time between the first and last half of the feeding trial in Experiment 1 (**g**) and 2 (**h**) are also displayed

There were no differences in the total feeding time (*t* = 0.12, *df* = 139.89, *p* = 0.901; Fig. 3a), the total food intake (*t* = − 1.07, *df* = 126.84, *p* = 0.286; Fig. 3b), the frequency of patch shifts (*t* = 0.77, *df* = 139.62, *p* = 0.443; Fig. 3c), and the mean speed of inter-patch movement (*t* = − 0.757, *df* = 126.78, *p* = 0.451; Fig. 3d) between Experiment 1 and 2. The mean residence time during the first half of the feeding trial did not differ between Experiment 1 and 2 (*t* = 1.00, *df* = 140.72, *p* = 0.321; Fig. 3e), but the mean residence time during the last half of the feeding trial (*t* = − 1.93, *df* = 118.08, *p* = 0.056; Fig. 3f) was significantly longer in Experiment 2. The mean residence time during the first half of the feeding trial was significantly longer than that during the last half of the feeding trial in both Experiment 1 (*t* = -7.52, *df* = 103.55, *p* < 0.001; Fig. 3g) and Experiment 2 (*t* = -4.52, *df* = 118.54,* p* < 0.001; Fig. 3h).

## Discussion

Like many other species [32, 33], the Japanese quails in this study behaved consistently in boldness and exploration (females: 0.363 < repeatability < 0.715; males: 0.556 < repeatability < 0.837) and the two behavioural traits were positively correlated (females: 0.905 < *r* < 1.000; males: 0.571 < *r* < 0.999), comprising a behavioural syndrome [29]. Although the correlation might be partly because the two behaviours were measured in the same arena, boldness and exploration have been found to be positively correlated in many species, i.e. bolder individuals exploring more in novel environments [34]. It has been found that these two personality traits have fitness consequences for animals through influencing important life-history decisions such as foraging strategies [12, 35]. The major finding of our study was that the two personality traits were related to food patch use of Japanese quail, that is, proactive (i.e. bolder and more explorative) quails had longer feeding time and more frequently changed food patches with less residence time in each food patch. The effect of personality on patch use also interacted with between-patch distances, weakening as the distance increased.

We found that proactive quails had longer feeding time and higher food intake in both experiments (Experiment 1 and 2). This finding was consistent with our previous study that the quails were allowed to feed in one food patch [29]. Previous studies have shown that proactive individuals have a faster pace-of-life with greater maximum metabolic rates than reactive ones [30]. More exploration might help proactive individuals find more food, motivating longer feeding time and resulting in higher food intake. The resultant higher food intake can meet the higher energetic requirements of proactive individuals. Although proactivity might increase animals’ risk of being preyed upon, it may benefit animals in obtaining more food [36–38]. For example, proactive great tits have preferential access to food because they have higher dominance than their reactive conspecifics in social foraging [39]. The higher energetic gains associated with the faster pace-of-life of proactive animals are suggested to offset the costs of movement and predation risks [27, 40].

Proactive quails had higher frequency of patch shifts and shorter residence time in each food patch when the two patches were nearer. In nature, food is usually patchily distributed and animals do not know in advance the food abundance in each patch. After animals enter a patch, the food availability of the patch would decline along with foraging [41]. When the food declines to a certain point, animals may not obtain expected amount of food given the same exploitation effort. They may leave the current patch to find new patches with potentially more food. However, animals have no complete information on the environment and would not be sure to find a patch with more food. In this case, they would face a trade-off between finding new patches (exploration) and continuing feeding in the current patch (exploitation: the exploration–exploitation trade-off) [26, 42]. The exploration–exploitation trade-off might vary among individuals with different personality traits, which was supported by our findings and Patrick et al. [26]. Because proactive individuals are more willing to take risks [26, 43], they may have more confidence in finding a patch with more food and thus leave the current patch earlier. In contrast, reactive (i.e. shyer and less explorative) individuals might be more uncertain in finding a new patch and are thus prone to stay longer in the current patch even though the food availability has now declined (Half a loaf is better than no bread) [44].

Many studies have found that inter-patch distances affect foraging behaviours among patches, such as prey consumption and residence time in food patches [45, 46]. In this study, the mean residence time during the first half of the feeding trial did not differ between experiments with different inter-patch distances. However, the quails reduced patch residence time along with feeding, and their mean residence time during the last half of the feeding trial in Experiment 2 was longer than that in Experiment 1. This indicated that the quails were more willing to leave the current patches along with satiety but this trend was weakened when the inter-patch distance was longer. Satiety may promote animals to explore new patches due to lowered risk of starvation, but the higher movement cost and difficulty of information gathering caused by longer inter-patch distances mediated the effect of satiety. More importantly, consistent with our expectation, the effect of personality traits on patch use varied with the between-patch distances [8, 22]. As discussed above, proactive quails more frequently changed patches and had shorter residence time in each patch when the two patches were nearer. But the effect disappeared when the inter-patch distance increased. The interaction between personality traits and between-patch distances on the patch use might be related to information gathering and movement cost. The difficulty of obtaining reliable information about the next food patches would increase with the between-patch distances [47]. Previous research has found that even a small increase in the distance can significantly reduce the capacity of ground-feeding birds to observe the next patch [48]. This would weaken animals' probability to find a patch of higher quality. In addition, the distance between patches is positively related to the movement cost, consuming more energy and increasing the risk of starvation [24]. Therefore, when food patches are far away, proactive animals might also less frequently change patches but instead spend more time searching in the current patch even though the food availability has reduced.

## Conclusions

We found that food patch use in domestic Japanese quails varied with the two personality traits (boldness and exploration). Proactive (i.e. bolder and more explorative) quails had longer feeding time and higher food intake, meeting their higher energy needs. Proactive individuals changed patches more frequently and had shorter residence time in each food patch when the patches were nearer, while the effects were not significant when the distance between patches increased. Besides, the quails reduced patch residence time along with feeding and this trend was weakened when the inter-patch distance was longer. Our study highlights the effect of personality traits on animals’ food patch use and its interaction with between-patch distances and therefore suggests that consistent inter-individual behavioural differences should be seriously considered when studying animals’ foraging behaviours.

## Methods

### Study subjects and breeding conditions

The domestic Japanese quails used in this study were from a farm in Changsha, China. All the quails on the farm were hatched and raised in batches according to Albus [49]. In each batch, over 2000 quails of mixed sexes were incubated for 16 days during which the temperature was 37 °C on the first day and then dropped by 0.5 °C every day until the room temperature (25 °C). After hatching, the new-borns were fed twice each day (i.e. 9 a.m. and 6 p.m.) under a natural 14/10 light/dark photoperiod. The basal diet for the quails contained corn (57%), soybean meal (30%), crude protein (5%), fish meal (5%), stone meal (2%), and soybean oil (1%). Sufficient water was provided for the quails, and maintenance was conducted at 8 a.m. each day.

When a batch of quails reached sexual maturity on the farm, we randomly selected a group of 6 healthy quails and transferred them to our laboratory. A total of 12 groups were consecutively selected from the farm from August to October 2020, resulting in 72 individuals used in our experiments. All subjects were approximately the same age (60 days) at the time of testing. In the laboratory, we housed the quails individually in labelled opaque cardboard containers (50 cm long, 40 cm wide, 60 cm high; hereafter referred to as housing container) under a natural photoperiod at 25 °C. A white ceramic tray (40 cm long, 35 cm wide, 3 cm high) sprinkled with sand was placed on the floor of each housing container for the quail to scratch and enjoy sand bathing [50]. There were two Petri dishes (9 cm diameter, 1.5 cm depth) on the tray, containing food and water separately. A white mesh was used to cover the top of the housing container to prevent the quail from escaping, while allowing cleaning of the tray and changing of Petri dishes. Daily cleaning was done at 8 a.m., and two daily feedings were given at 9 a.m. and 6 p.m.

To acclimate to the laboratory conditions, the quails were placed in the housing container for three days before the experiments. During the acclimatization and experiments, small yellow millets (MILLET, Wuchang Rice Products Co., Ltd., Wuhan, China; 18% energy, 17% protein, 6% lipid, 24% carbohydrates) sieved to consistent size (diameter: 1 mm; grain weight: 0.002 g) were used as food for the quails. Once the experiments started, the quails were only fed ad libitum during and immediately after the trials. To ensure that the quails were food-motivated during experiments, we did not feed them 24 h before the trials.

### General experimental process

**Fig. 4:**
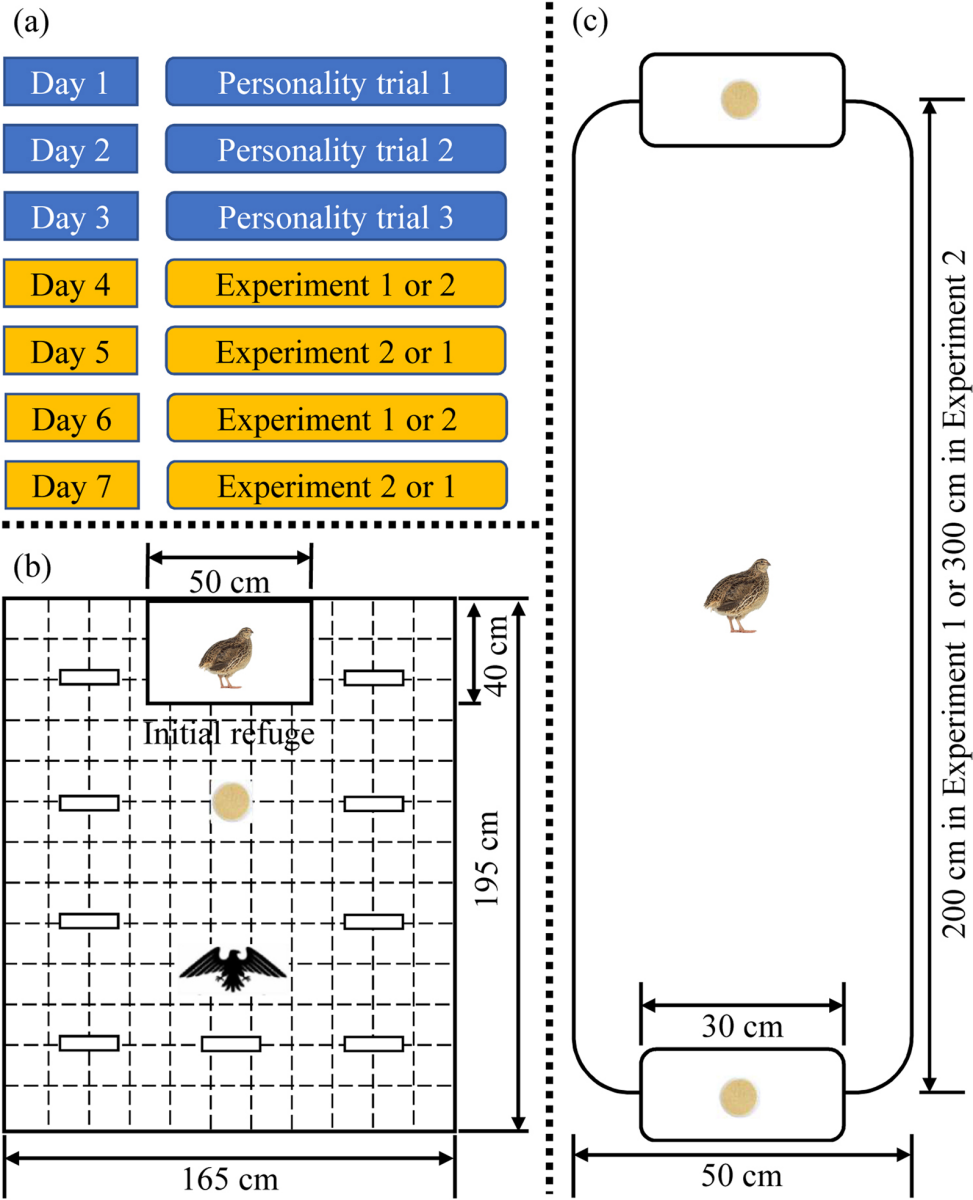
Overview diagram of the trials (**a**), top view of the open arena (**b**) and foraging ground in the patch use experiments (**c**)

Firstly, boldness and exploration for each quail were measured three times on successive days using open arena assays (see the subsection of Personality trials for details) to test behavioural repeatability (Fig. 4a). Subsequently, the pattern of food patch use with different inter-patch distances (i.e. 2 m in Experiment 1 and 3 m in Experiment 2) was quantified twice for each subject during foraging experiments (see the subsection of Patch use trials for details). We randomized the trial orders of the quails, and maintained the same ambient conditions (i.e. quiet with no disturbances) throughout the experiments in the same laboratory. During the trials, the experimenters were shielded from the subjects by a 1.5-m high opaque curtain to avoid potential disturbances. To minimize observer bias, blinded methods were used when all behavioural data were recorded and/or analysed. At the end of the experiments for each batch of quails, all subjects were weighed to 0.1 g.

### Personality trials

The open arena used to measure boldness and exploration was a rectangular field (195 cm long, 165 cm wide, 150 cm high) surrounded by grey opaque curtains (Fig. 1 in Zhang et al. [29] and Fig. 4b in this study). The ground of the arena was divided into 143 squares (15 cm × 15 cm) by dark lines. There was a camera (Sony HDR-CX510, 55 × extended zooms, Sony Corporation, Tokyo, Japan) over the arena to monitor the tested quails. One end of the arena was connected to an initial refuge (a different box but with the same dimensions as the housing container) which had a sliding trapdoor (15 cm × 15 cm) facing the arena. The experimenter could remotely open the sliding trapdoor by pulling a fishing line, allowing the subject to walk from the initial refuge to the arena. A Petri dish (the same as that in the housing container) containing 20 artificial leaves (red and green; about 3 cm long and 2 cm wide; covering the food to simulate buried food resources in the wild) was placed 30 cm in front of the trapdoor. To complicate the environment, the arena was equipped with nine novel objects (standing cardboard; 20 cm long, 10 cm high). To maintain novelty of the objects, the standing cardboards were different colours, i.e. white, red, and blue, respectively, in the three repeated trials. To make the same experimental conditions for each quail, the locations of the standing cardboards were maintained for different subjects. Following Quinn and Cresswell [51], we used a hawk model (26 cm wingspan × 6 cm high, and weighs approximately 50 g) to simulate the risk of predation for the quails. The hawk model was hung by a fishing line, 90 cm in front of the trapdoor, and the experimenter could pull the model from the ground to a height of 1.5 m out of the sight of the subjects.

At the beginning of the personality trials, we randomly selected a quail and gently transferred it to the initial refuge, and then turned on the camera. The quail was given 5 min to acclimate to the initial refuge before the experimenter remotely opened the trapdoor. Immediately after the trapdoor was opened, the experimenter remotely pulled the hawk model from the ground to a height of 10 cm at a constant speed (about 10 cm/s). The model was hung at 10 cm above the ground until the subject walked out of the refuge, after which the model was slowly pulled up and placed at a height of 1.5 m out of sight of the subject. We gave each subject a maximum of 20 min to walk out of the initial refuge, and defined boldness of the subject as 20 min minus the time taken to emerge [52]. The quail was considered to have emerged from the initial refuge when its whole body crossed the trapdoor. The movement of the subject was continuously recorded by the camera for 12 min after it entered the arena. If the subject did not emerge within the given time, its boldness score was determined as 0 s, i.e. 20 min minus 20 min. In this case, the quail was gently moved by the experimenter from the refuge to the arena, and was continuously monitored by the camera for 12 min. The last 10 min for each subject in the arena was referred as its exploration trial. After the trials, 600 image stacks (one frame per second) were extracted from the 10-min exploration videos and Image J (http://rsbweb.nih.gov/ij/) was used to delineate the movement of the quail. Similar to Bousquet et al. [53], the exploration score of each subject was determined as the total number of squares that the subject passed without repetitions. Following Sih et al. (2004), we here referred exploration to “activity in an unfamiliar environment”. Furthermore, we used the last 10 min of the 12-min video of movement after the subject entered the arena. The 2-min interval may help to disentangle boldness and exploration.

### Patch use trials

Patch use trials were carried out in a rectangular field (hereafter, foraging ground) which had fixed width (50 cm) and height (150 cm) but changeable length (200 cm for Experiment 1 and 300 cm for Experiment 2; Fig. 4c). Japanese quail is a shy, ground-feeding bird species of small size (20 cm) and even small increases in inter-patch distances may significantly reduce its capacity of observing next patches. In our pilot experiments, we found that the quails rarely changed patches when the inter-patch distance was longer than 3 m. Therefore, we conducted patch use trials with inter-patch distances of 2 m and 3 m. The order of the trials was Experiment 1–2-1–2 or 2–1–2–1 (randomly for subjects) in four successive days (Fig. 4a). Above the foraging ground, there was a camera (Sony HDR-CX510, 55 × extended zooms, Sony Corporation, Tokyo, Japan) used to monitor the quail. At each end of the foraging ground, there was a piece of hardboard (30 cm long and 20 cm wide) on which a Petri dish (the same as that in the housing container) was placed to be used as a food patch. A pile of small yellow millet (2 g) was put in the Petri dish, with 10 artificial leaves (the same as those in the personality trials) covering the food. There was one experimenter at each end of the foraging ground, hiding behind the curtain and preparing to change the Petri dishes.

At the beginning of the trials, the camera was turned on. The experimenters gently transferred a quail from the housing container to the centre of the foraging ground and monitored the subject through the camera screen from a distance. The quail could see and was familiar with the food patch at the two ends of the foraging ground, but it could not see the food in the Petri dish. The experiments started when the quail began to search for food at one patch. When the subject left the current patch and arrived the other patch, one experimenter gently replaced the current Petri dish with a new one which had the same amount of buried food. The replaced dishes were labelled with their orders of being visited by the subject and the remaining food in each Petri dish was weighed after the trial. Each patch use trial lasted for 40 min when most quails stopped feeding (observed in pilot experiments). After the trial, the quail was transferred back to its housing container and the foraging ground and the patches were cleaned to remove any scent trails.

Through video playback, we recorded the following data for each subject during each foraging trial: (1) total number of food patches visited during the trial; (2) the weight (g) of food obtained in each food patch; (3) visiting order and time when visiting each food patch; (4) time when leaving each patch; (5) residence time (s) in each patch; (6) traveling time (s) between two patches; (7) time (s) that the individual was immobile between patches. Based on these data, we quantified the following patch use variables for each individual to be used in the subsequent analyses: (1) total feeding time (TFT; the total trial time—the time that the individual was significantly stagnant between patches—the total traveling time between patches; TFT might be shorter than the sum of residence time because subjects may not feed while staying in a food patch); (2) total food intake (TFI; the sum of the food weight obtained in all the food patches); (3) frequency of patch shifts (FPS; the number of visited patches—1); (4) mean speed of movement between two patches (MSM). Considering the influence of satiety on the residence time in each patch, we calculated two means of residence time in food patches: (5) the mean residence time during the first half of the feeding trial (MRF) and (6) the mean residence time during the last half of the feeding trial (MRL). If a subject did not change food patch during the trials, we recorded the fourth and sixth variable as “NA”.

### Statistical analyses

Zhang et al. analysed the same data of boldness and exploration and found that the two behaviours were significantly repeatable and constituted a behavioural syndrome [29]. In this study, the two behavioural traits for each subject were averaged from the three personality trials, and the six patch use variables were averaged from the two patch use trials for each inter-patch distance. We used the Shapiro–Wilk Test to examine the normality of these means and found they were normally distributed (*p* > 0.05) and therefore we did not transform these means in the following analyses. However, the residence time in each food patch was not normally distributed and was log_10_ transformed to be used. Because the two personality traits were strongly correlated [29], a principal component analysis (PCA) was performed using the R package *psych* and we obtained two new variables that were orthogonally rotated: the principal components 1 and 2 (PC1 and PC2). PC1 (loadings: 0.707 boldness + 0.707 exploration; eigenvalue: 1.296) and PC2 (loadings: − 0.707 exploration + 0.707 boldness; eigenvalue: 0.565) explained 84.0% and 16.0% of the total variance, respectively [29]. We labelled PC1 as “proactivity” in this study and did not consider PC2 in the following GLMs because of its low eigenvalue and variance explained.

The function *corr.test* in the package *psych* [54] was used to calculate the Pearson correlation coefficient between the residence time in each food patch (log_10_ transformed) and its visiting order. Individual general linear models (GLMs) were used to test how the six foraging variables were affected by proactivity, as well as body weight and sex which might also be correlated with foraging behaviours. We initially included the two-way interactions between body weight, sex, and proactivity, but excluded them from the final models because of no significant effects. *T*-tests were used to examine the differences in the six foraging variables between Experiment 1 and 2. All statistical analyses were performed with R 3.6.3 (R Development Core Team 2019), and the data are displayed as mean ± standard error (SE).

## Data Availability

All data in this study are provided as supplementary materials.
